# Supplementary material for: Integrative Map of HIF1A Regulatory Elements and Variations
Source: Genes (Basel). 2021 Sep 28;12(10):1526. doi: 10.3390/genes12101526 (PMC8536025; doi:10.3390/genes12101526)
Supplement: Supplementary file 1 [file genes-12-01526-s001.zip › genes-1369065-supplementary.pdf]

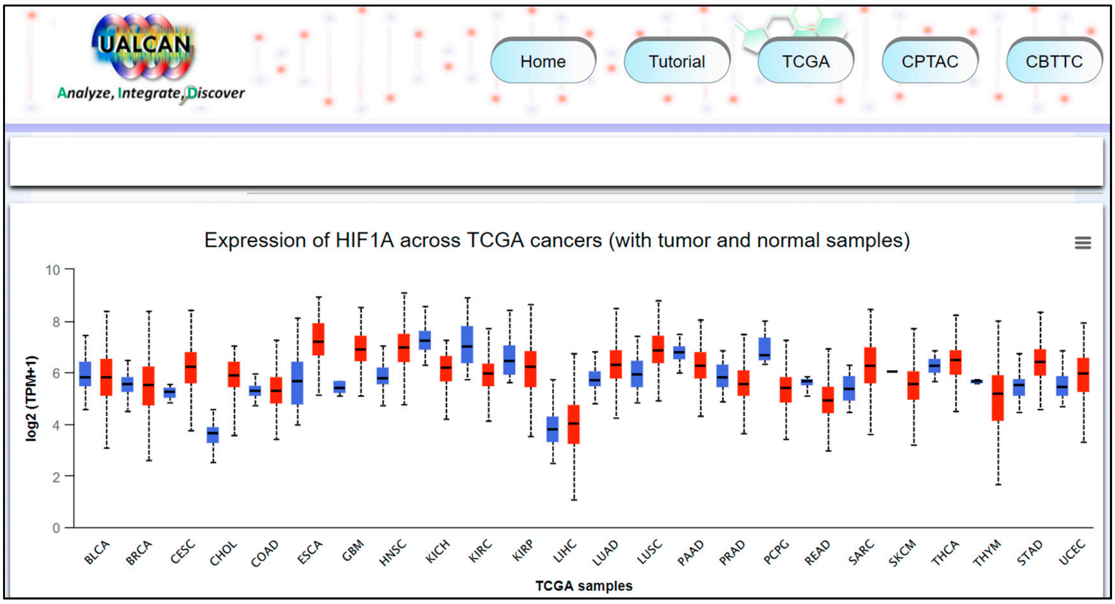
UALCAN
Analyze, Integrate, Discover
Home
Tutorial
TCGA
CPTAC
CBTTC
Expression of HIF1A across TCGA cancers (with tumor and normal samples)
log2 (TPM+1)
10
8
6
4
2
0
BLCA
BRCA
CESC
CHOL
COAD
ESCA
GBM
HNSC
KICH
KIRC
KIRP
LIHC
LUAD
LUSC
PAAD
PRAD
PCPG
READ
SARC
SKCM
THCA
THYM
STAD
UCEC
TCGA samples

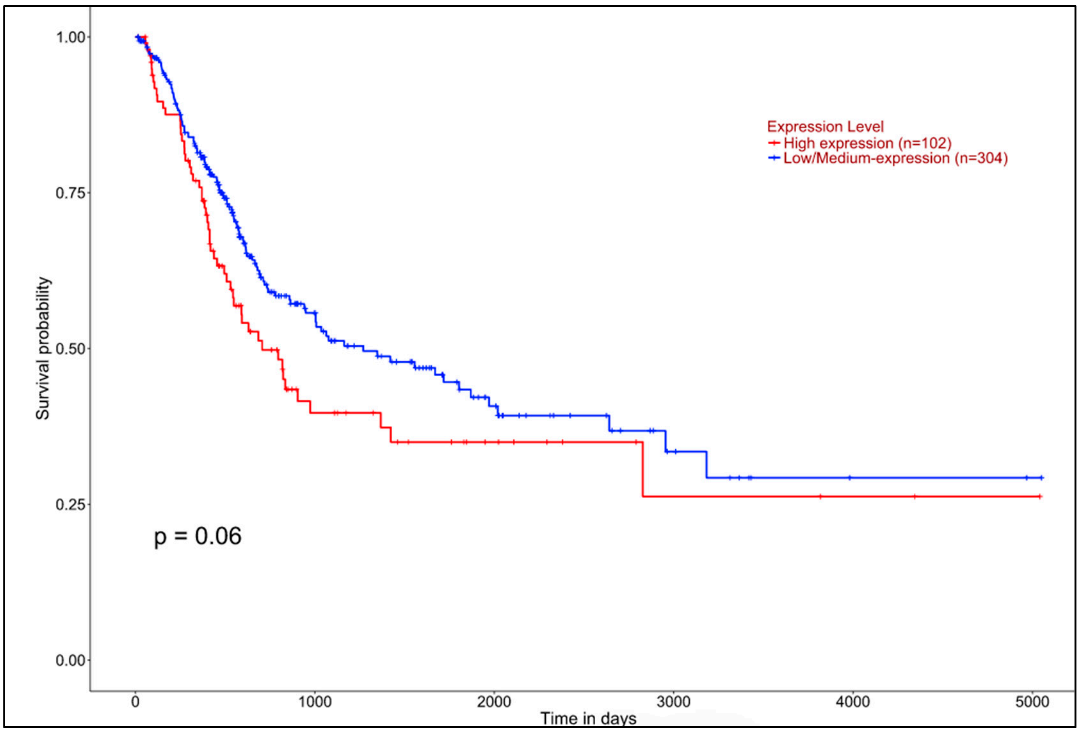
1.00
0.75
0.50
0.25
0.00
Survival probability
Expression Level
High expression (n=102)
Low/Medium-expression (n=304)
p = 0.06
0
1000
2000
3000
4000
5000
Time in days

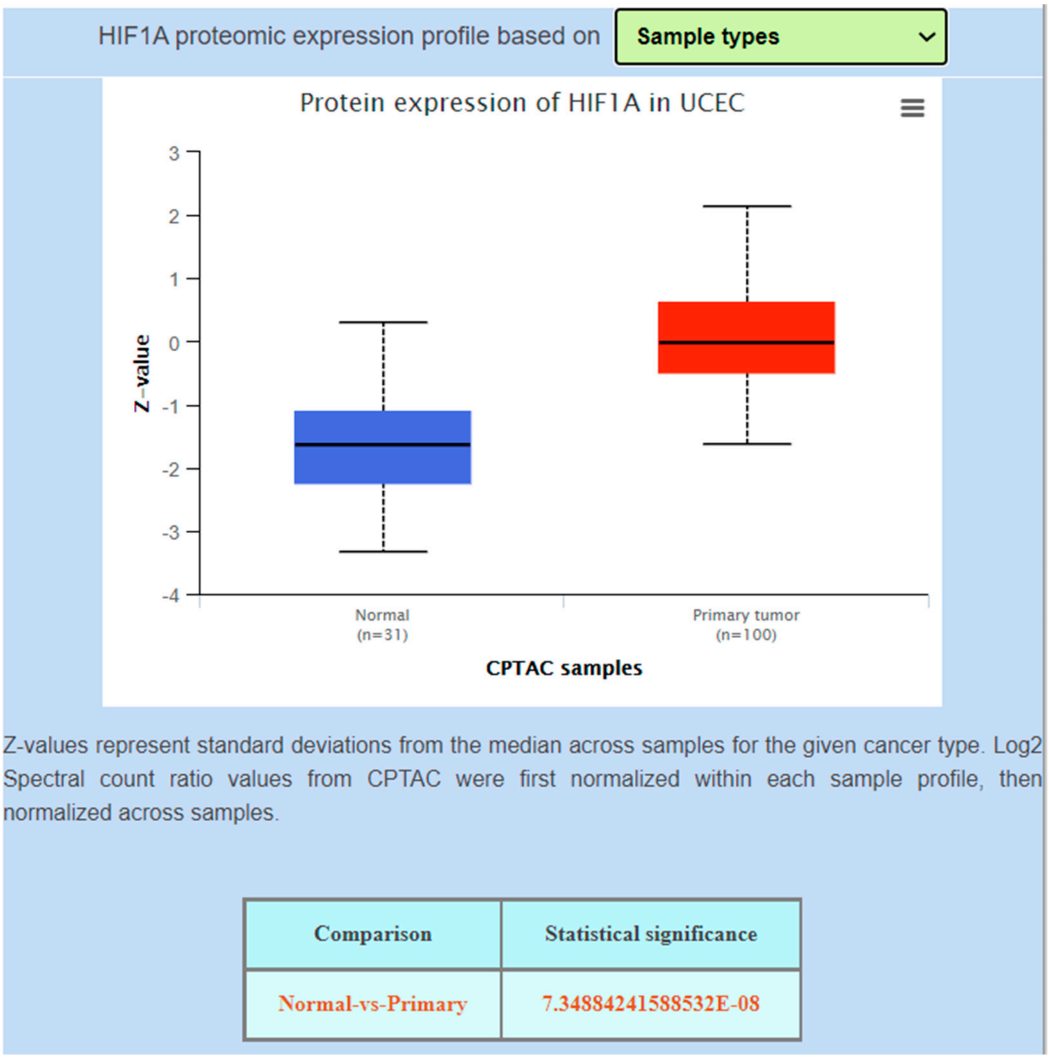

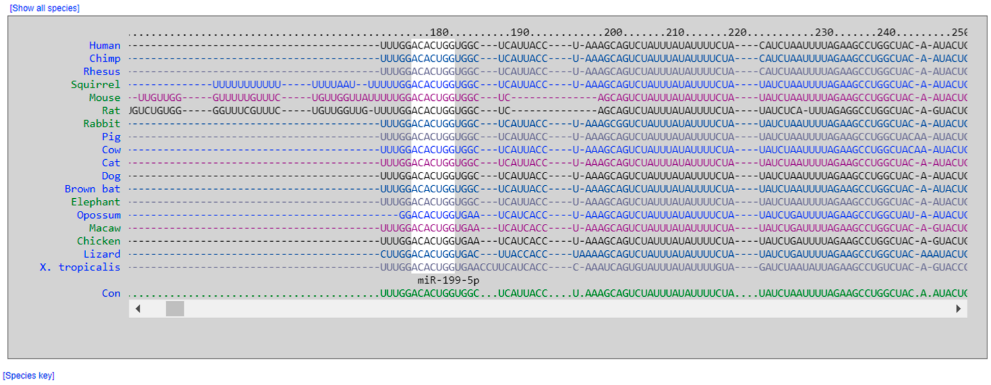

[Download table]

Conserved

	Predicted consequential pairing of target region (top) and miRNA (bottom)	Site type	Context++ score	Context++ score percentile	Weighted context++ score	Conserved branch length	P_{CT}
Position 177-183 of HIF1A 3' UTR<br>hsa-miR-199b-5p	5' ...CAUUCCUUUUUUUGGACACUGGU...<br>||||||| 3' CUUGUCUAUCAGAUUUGUGACCC	7mer-m8	-0.22	91	-0.22	9.367	> 0.99
Position 177-183 of HIF1A 3' UTR<br>hsa-miR-199a-5p	5' ...CAUUCCUUUUUUUGGACACUGGU...<br>||||||| 3' CUUGUCCAUCAGACUUGUGACCC	7mer-m8	-0.23	91	-0.23	9.367	> 0.99

Figure S1: *HIF1A* data, extracted from the UALCAN database, an interactive web resource for analyzing cancer OMICS data. **A.** Expression of *HIF1A* across TCGA cancers with tumor and normal samples. **B**: Effect of *HIF1A* expression level on bladder urothelial carcinoma (BLCA) patient survival. **C**: HIF1A proteomic expression profile based on sample types. **D.** Conserved miRNA mir-199-5p target site within the 3' UTR region of the *HIF1A* gene.

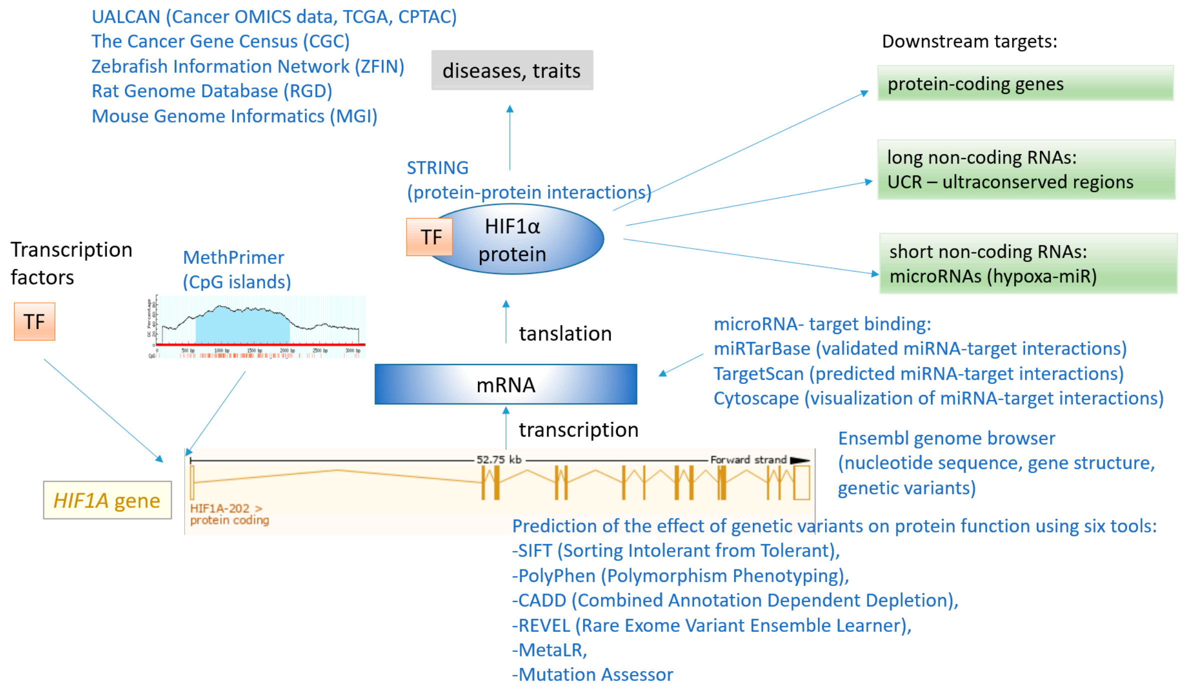

Figure S2: Summary of genomic databases and bioinformatics tools used in the present analysis.

Table S1. Phenotypes, diseases and traits associated with the *HIF1A* gene in human. Data obtained from the Ensembl genome browser.

Acute myeloid leukemia	Cancer Gene Census
Adrenal Gland Neuroblastoma	Cancer Gene Census
Ampulla of Vater Carcinoma	Cancer Gene Census
Atypical Meningioma	Cancer Gene Census
Basal cell carcinoma	Cancer Gene Census
bile duct carcinoma	Cancer Gene Census
bladder carcinoma	Cancer Gene Census
brain glioblastoma	Cancer Gene Census
Breast carcinoma	Cancer Gene Census
breast ductal adenocarcinoma	Cancer Gene Census
bronchoalveolar adenocarcinoma	Cancer Gene Census
cecum adenocarcinoma	Cancer Gene Census
Cecum Villous Adenoma	Cancer Gene Census
cervical squamous cell carcinoma	Cancer Gene Census
Chronic lymphocytic leukemia	Cancer Gene Census
Clear cell renal carcinoma	Cancer Gene Census
colon adenocarcinoma	Cancer Gene Census
colorectal adenocarcinoma	Cancer Gene Census
Cutaneous melanoma	Cancer Gene Census
diffuse large B-cell lymphoma	Cancer Gene Census
Endometrial carcinoma	Cancer Gene Census
Endometrial Endometrioid Adenocarcinoma	Cancer Gene Census

Esophageal adenocarcinoma	Cancer Gene Census
Esophageal Squamous Cell Carcinoma	Cancer Gene Census
gastric adenocarcinoma	Cancer Gene Census
Gastric Adenoma	Cancer Gene Census
gastric carcinoma	Cancer Gene Census
head and neck squamous cell carcinoma	Cancer Gene Census
Hepatobiliary Neoplasm	Cancer Gene Census
Hepatocellular Carcinoma	Cancer Gene Census
HER2 Positive Breast Carcinoma	Cancer Gene Census
Invasive Breast Carcinoma	Cancer Gene Census
kidney neoplasm	Cancer Gene Census
Kidney Oncocytoma	Cancer Gene Census
lobular breast carcinoma	Cancer Gene Census
Lung adenocarcinoma	Cancer Gene Census
Lung carcinoma	Cancer Gene Census
lymphoid neoplasm	Cancer Gene Census
mantle cell lymphoma	Cancer Gene Census
Merkel cell skin cancer	Cancer Gene Census
Multiple myeloma	Cancer Gene Census
nasopharyngeal squamous cell carcinoma	Cancer Gene Census
non-functional pancreatic neuroendocrine tumor	Cancer Gene Census
non-small cell lung carcinoma	Cancer Gene Census
ovarian endometrioid adenocarcinoma with squamous differentiation	Cancer Gene Census
ovarian serous adenocarcinoma	Cancer Gene Census
pancreatic ductal adenocarcinoma	Cancer Gene Census
pancreatic neuroendocrine tumor	Cancer Gene Census
Papillary renal cell carcinoma	Cancer Gene Census
prostate adenocarcinoma	Cancer Gene Census
prostate carcinoma	Cancer Gene Census
rectal adenocarcinoma	Cancer Gene Census
rectal tubulovillous adenoma	Cancer Gene Census
Rhabdoid Meningioma	Cancer Gene Census
skin carcinoma	Cancer Gene Census
Small cell lung carcinoma	Cancer Gene Census
soft tissue sarcoma	Cancer Gene Census
squamous cell lung carcinoma	Cancer Gene Census
thyroid carcinoma	Cancer Gene Census
Thyroid Gland Undifferentiated (Anaplastic) Carcinoma	Cancer Gene Census
Uterine Carcinosarcoma	Cancer Gene Census
Exercise Test	dbGaP
Uric Acid	dbGaP
Malignant tumor of prostate	ClinVar
Blood pressure	dbGaP

Table S2. Phenotypes, diseases and traits associated with the *HIF1A* gene in animals. Data obtained from the Ensembl genome browser.

Phenotype, disease and trait	Source	Species	Gene
whole organism, increased amount	ZFIN	Zebrafish	ENSDARG00000034293
			hif1ab
whole organism, decreased amount	ZFIN	Zebrafish	ENSDARG00000006181
			hif1aa
whole organism, decreased amount	ZFIN	Zebrafish	ENSDARG00000034293
			hif1ab
Werner syndrome	RGD	Rat	ENSRNOG00000008292
			Hif1a
Varicocele	RGD	Rat	ENSRNOG00000008292
			Hif1a
Urologic neoplasms	RGD	Rat	ENSRNOG00000008292
			Hif1a
Ulcer	RGD	Rat	ENSRNOG00000008292
			Hif1a
type 2 diabetes mellitus	RGD	Rat	ENSRNOG00000008292
			Hif1a
transient cerebral ischemia	RGD	Rat	ENSRNOG00000008292
			Hif1a
Thyroid neoplasms	RGD	Rat	ENSRNOG00000008292
			Hif1a
Testis Reperfusion Injury	RGD	Rat	ENSRNOG00000008292
			Hif1a
Temporomandibular Joint Disorders	RGD	Rat	ENSRNOG00000008292
			Hif1a
Subarachnoid Hemorrhage	RGD	Rat	ENSRNOG00000008292
			Hif1a
splanchnocranium, morphology	ZFIN	Zebrafish	ENSDARG00000034293
			hif1ab
splanchnocranium, malformed	ZFIN	Zebrafish	ENSDARG00000034293
			hif1ab
Spinal Cord Injuries	RGD	Rat	ENSRNOG00000008292
			Hif1a
small second pharyngeal arch	MGI	Mouse	ENSMUSG00000021109
			Hif1a
Skin neoplasms	RGD	Rat	ENSRNOG00000008292
			Hif1a
skin melanoma	RGD	Rat	ENSRNOG00000008292
			Hif1a
sclerosing cholangitis	RGD	Rat	ENSRNOG00000008292
			Hif1a
rheumatoid arthritis	RGD	Rat	ENSRNOG00000008292
			Hif1a
Retinal neovascularization	RGD	Rat	ENSRNOG00000008292
			Hif1a
Retinal Ischemia	RGD	Rat	ENSRNOG00000008292

			Hif1a
retina, increased amount	ZFIN	Zebrafish	ENSDARG00000034293<br>hif1ab
respiratory gaseous exchange by respiratory system, increased frequency	ZFIN	Zebrafish	ENSDARG00000034293<br>hif1ab
Reperfusion Injury	RGD	Rat	ENSRNOG00000008292<br>Hif1a
renal cell carcinoma	RGD	Rat	ENSRNOG00000008292<br>Hif1a
Radiation Injuries, Experimental	RGD	Rat	ENSRNOG00000008292<br>Hif1a
Radiation Injuries	RGD	Rat	ENSRNOG00000008292<br>Hif1a
pulmonary hypertension	RGD	Rat	ENSRNOG00000008292<br>Hif1a
psoriasis	RGD	Rat	ENSRNOG00000008292<br>Hif1a
Prostatic neoplasms	RGD	Rat	ENSRNOG00000008292<br>Hif1a
prostate cancer	RGD	Rat	ENSRNOG00000008292<br>Hif1a
primary biliary cirrhosis	RGD	Rat	ENSRNOG00000008292<br>Hif1a
primary biliary cholangitis	RGD	Rat	ENSRNOG00000008292<br>Hif1a
prenatal lethality, incomplete penetrance	MGI	Mouse	ENSMUSG00000021109<br>Hif1a
prenatal lethality, complete penetrance	MGI	Mouse	ENSMUSG00000021109<br>Hif1a
Postmenopausal Osteoporosis	RGD	Rat	ENSRNOG00000008292<br>Hif1a
polycystic kidney disease	RGD	Rat	ENSRNOG00000008292<br>Hif1a
Phyllodes tumor	RGD	Rat	ENSRNOG00000008292<br>Hif1a
pharyngeal arch artery hypoplasia	MGI	Mouse	ENSMUSG00000021109<br>Hif1a
pericardial effusion	MGI	Mouse	ENSMUSG00000021109<br>Hif1a
pancreatic cancer	RGD	Rat	ENSRNOG00000008292<br>Hif1a
Oxygen-Induced Retinopathy	RGD	Rat	ENSRNOG00000008292<br>Hif1a
ovarian cancer	RGD	Rat	ENSRNOG00000008292<br>Hif1a
Osteoarthritis	RGD	Rat	ENSRNOG00000008292<br>Hif1a

optic cup, increased amount	ZFIN	Zebrafish	ENSDARG00000034293
			hif1ab
open neural tube	MGI	Mouse	ENSMUSG00000021109
			Hif1a
olfactory neuroblastoma	RGD	Rat	ENSRNOG00000008292
			Hif1a
non-small cell lung carcinoma	RGD	Rat	ENSRNOG00000008292
			Hif1a
no abnormal phenotype detected	MGI	Mouse	ENSMUSG00000021109
			Hif1a
neural crest cell migration, decreased process quality	ZFIN	Zebrafish	ENSDARG00000034293
			hif1ab
neural crest cell migration, decreased occurrence	ZFIN	Zebrafish	ENSDARG00000034293
			hif1ab
nephroblastoma	RGD	Rat	ENSRNOG00000008292
			Hif1a
Neoplastic Cell Transformation	RGD	Rat	ENSRNOG00000008292
			Hif1a
Neoplasm Invasiveness	RGD	Rat	ENSRNOG00000008292
			Hif1a
Myocardial Reperfusion Injury	RGD	Rat	ENSRNOG00000008292
			Hif1a
Myocardial Ischemia	RGD	Rat	ENSRNOG00000008292
			Hif1a
Myocardial Infarction	RGD	Rat	ENSRNOG00000008292
			Hif1a
Mycosis Fungoides	RGD	Rat	ENSRNOG00000008292
			Hif1a
motor neuron, part_of, trunk, increased amount	ZFIN	Zebrafish	ENSDARG00000034293
			hif1ab
morbid obesity	RGD	Rat	ENSRNOG00000008292
			Hif1a
middle cerebral artery infarction	RGD	Rat	ENSRNOG00000008292
			Hif1a
Micronuclei, Chromosome-Defective	RGD	Rat	ENSRNOG00000008292
			Hif1a
Memory Disorders	RGD	Rat	ENSRNOG00000008292
			Hif1a
Mammary Neoplasms, Experimental	RGD	Rat	ENSRNOG00000008292
			Hif1a
lung non-small cell carcinoma	RGD	Rat	ENSRNOG00000008292
			Hif1a
lung disease	RGD	Rat	ENSRNOG00000008292
			Hif1a
Limb Ischemia	RGD	Rat	ENSRNOG00000008292
			Hif1a
Kidney Reperfusion Injury	RGD	Rat	ENSRNOG00000008292

			Hif1a
kidney failure	RGD	Rat	ENSRNOG00000008292<br>Hif1a
invasive ductal carcinoma	RGD	Rat	ENSRNOG00000008292<br>Hif1a
Intervertebral Disc Disease	RGD	Rat	ENSRNOG00000008292<br>Hif1a
infarction	RGD	Rat	ENSRNOG00000008292<br>Hif1a
increased tumor necrosis factor secretion	MGI	Mouse	ENSMUSG00000021109<br>Hif1a
increased interferon-gamma secretion	MGI	Mouse	ENSMUSG00000021109<br>Hif1a
increased hindbrain size	MGI	Mouse	ENSMUSG00000021109<br>Hif1a
increased embryonic tissue cell apoptosis	MGI	Mouse	ENSMUSG00000021109<br>Hif1a
increased apoptosis	MGI	Mouse	ENSMUSG00000021109<br>Hif1a
incomplete somite formation	MGI	Mouse	ENSMUSG00000021109<br>Hif1a
Idiopathic Pulmonary Fibrosis	RGD	Rat	ENSRNOG00000008292<br>Hif1a
Hypoxia-Ischemia, Brain	RGD	Rat	ENSRNOG00000008292<br>Hif1a
hypoxia	MGI	Mouse	ENSMUSG00000021109<br>Hif1a
hypertension	RGD	Rat	ENSRNOG00000008292<br>Hif1a
Hyperplasia	RGD	Rat	ENSRNOG00000008292<br>Hif1a
Hyperalgesia	RGD	Rat	ENSRNOG00000008292<br>Hif1a
Hot Flashes	RGD	Rat	ENSRNOG00000008292<br>Hif1a
hepatocellular carcinoma	RGD	Rat	ENSRNOG00000008292<br>Hif1a
Hepatic Encephalopathy	RGD	Rat	ENSRNOG00000008292<br>Hif1a
hemorrhage	MGI	Mouse	ENSMUSG00000021109<br>Hif1a
hematopoietic stem cell, part_of, ventral wall of dorsal aorta, decreased amount	ZFIN	Zebrafish	ENSDARG00000034293<br>hif1ab
Hemangioblastoma	RGD	Rat	ENSRNOG00000008292<br>Hif1a
Hearing Loss, Noise-Induced	RGD	Rat	ENSRNOG00000008292<br>Hif1a

head, increased amount	ZFIN	Zebrafish	ENSDARG00000034293<br>hif1ab
goblet cell, part_of, intestine, decreased amount	ZFIN	Zebrafish	ENSDARG00000034293<br>hif1ab
glycogen storage disease VII	RGD	Rat	ENSRNOG00000008292<br>Hif1a
glycogen storage disease V	RGD	Rat	ENSRNOG00000008292<br>Hif1a
glioblastoma multiforme	RGD	Rat	ENSRNOG00000008292<br>Hif1a
glioblastoma	RGD	Rat	ENSRNOG00000008292<br>Hif1a
Femur Head Necrosis	RGD	Rat	ENSRNOG00000008292<br>Hif1a
Experimental Neoplasms	RGD	Rat	ENSRNOG00000008292<br>Hif1a
Experimental Diabetes Mellitus	RGD	Rat	ENSRNOG00000008292<br>Hif1a
Experimental Autoimmune Neuritis	RGD	Rat	ENSRNOG00000008292<br>Hif1a
Experimental Arthritis	RGD	Rat	ENSRNOG00000008292<br>Hif1a
esophagus squamous cell carcinoma	RGD	Rat	ENSRNOG00000008292<br>Hif1a
embryonic lethality during organogenesis, incomplete penetrance	MGI	Mouse	ENSMUSG00000021109<br>Hif1a
embryonic lethality during organogenesis, complete penetrance	MGI	Mouse	ENSMUSG00000021109<br>Hif1a
embryonic lethality between implantation and somite formation, incomplete penetrance	MGI	Mouse	ENSMUSG00000021109<br>Hif1a
embryonic growth retardation	MGI	Mouse	ENSMUSG00000021109<br>Hif1a
embryonic growth arrest	MGI	Mouse	ENSMUSG00000021109<br>Hif1a
ductal carcinoma in situ	RGD	Rat	ENSRNOG00000008292<br>Hif1a
Ductal Carcinoma	RGD	Rat	ENSRNOG00000008292<br>Hif1a
disorganized yolk sac vascular plexus	MGI	Mouse	ENSMUSG00000021109<br>Hif1a
disorganized myocardium	MGI	Mouse	ENSMUSG00000021109<br>Hif1a
Disease Progression	RGD	Rat	ENSRNOG00000008292<br>Hif1a
dilated dorsal aorta	MGI	Mouse	ENSMUSG00000021109<br>Hif1a

Diabetic Retinopathy	RGD	Rat	ENSRNOG00000008292
			Hif1a
Diabetic Nephropathies	RGD	Rat	ENSRNOG00000008292
			Hif1a
Diabetes Mellitus, Experimental	RGD	Rat	ENSRNOG00000008292
			Hif1a
Diabetes Complications	RGD	Rat	ENSRNOG00000008292
			Hif1a
decubitus ulcer	RGD	Rat	ENSRNOG00000008292
			Hif1a
decreased neuron apoptosis	MGI	Mouse	ENSMUSG00000021109
			Hif1a
decreased embryo size	MGI	Mouse	ENSMUSG00000021109
			Hif1a
decreased angiogenesis	MGI	Mouse	ENSMUSG00000021109
			Hif1a
congestive heart failure	RGD	Rat	ENSRNOG00000008292
			Hif1a
congenital heart disease	RGD	Rat	ENSRNOG00000008292
			Hif1a
Colonic Neoplasms	RGD	Rat	ENSRNOG00000008292
			Hif1a
Chronic Cerebral Hypoperfusion	RGD	Rat	ENSRNOG00000008292
			Hif1a
cholesteatoma of middle ear	RGD	Rat	ENSRNOG00000008292
			Hif1a
Cell Transformation, Neoplastic	RGD	Rat	ENSRNOG00000008292
			Hif1a
cell maturation, occurs_in, goblet cell, disrupted	ZFIN	Zebrafish	ENSDARG00000034293
			hif1ab
Cardiomegaly	RGD	Rat	ENSRNOG00000008292
			Hif1a
cardia bifida	MGI	Mouse	ENSMUSG00000021109
			Hif1a
Carcinoma	RGD	Rat	ENSRNOG00000008292
			Hif1a
Burns	RGD	Rat	ENSRNOG00000008292
			Hif1a
Bronchopulmonary Dysplasia	RGD	Rat	ENSRNOG00000008292
			Hif1a
Breast Neoplasms	RGD	Rat	ENSRNOG00000008292
			Hif1a
Brain Ischemia	RGD	Rat	ENSRNOG00000008292
			Hif1a
bladder neck obstruction	RGD	Rat	ENSRNOG00000008292
			Hif1a
axon, part_of, motor neuron, truncated	ZFIN	Zebrafish	ENSDARG00000034293

			hif1ab
axon guidance, occurs_in, motor neuron, decreased process quality	ZFIN	Zebrafish	ENSDARG00000034293
			hif1ab
Atrial Fibrillation	RGD	Rat	ENSRNOG00000008292
			Hif1a
Astrocytoma	RGD	Rat	ENSRNOG00000008292
			Hif1a
asthma	RGD	Rat	ENSRNOG00000008292
			Hif1a
Asphyxia	RGD	Rat	ENSRNOG00000008292
			Hif1a
Arterial Thrombosis	RGD	Rat	ENSRNOG00000008292
			Hif1a
Anaplasia	RGD	Rat	ENSRNOG00000008292
			Hif1a
Alzheimer's Disease	RGD	Rat	ENSRNOG00000008292
			Hif1a
Alcoholic Liver Diseases	RGD	Rat	ENSRNOG00000008292
			Hif1a
Acute-Phase Reaction	RGD	Rat	ENSRNOG00000008292
			Hif1a
acute kidney failure	RGD	Rat	ENSRNOG00000008292
			Hif1a
absent third pharyngeal arch	MGI	Mouse	ENSMUSG00000021109
			Hif1a
absent second pharyngeal arch	MGI	Mouse	ENSMUSG00000021109
			Hif1a
absent pharyngeal arch arteries	MGI	Mouse	ENSMUSG00000021109
			Hif1a
absent myocardial trabeculae	MGI	Mouse	ENSMUSG00000021109
			Hif1a
abnormal vitelline vascular remodeling	MGI	Mouse	ENSMUSG00000021109
			Hif1a
abnormal vascular regression	MGI	Mouse	ENSMUSG00000021109
			Hif1a
abnormal vascular development	MGI	Mouse	ENSMUSG00000021109
			Hif1a
abnormal somite development	MGI	Mouse	ENSMUSG00000021109
			Hif1a
abnormal pharyngeal arch morphology	MGI	Mouse	ENSMUSG00000021109
			Hif1a
abnormal pericardium morphology	MGI	Mouse	ENSMUSG00000021109
			Hif1a
abnormal pericardial cavity morphology	MGI	Mouse	ENSMUSG00000021109
			Hif1a
abnormal neural fold formation	MGI	Mouse	ENSMUSG00000021109
			Hif1a

abnormal neural crest cell migration	MGI	Mouse	ENSMUSG00000021109
			Hif1a
abnormal myocardium layer morphology	MGI	Mouse	ENSMUSG00000021109
			Hif1a
abnormal myocardial trabeculae morphology	MGI	Mouse	ENSMUSG00000021109
			Hif1a
abnormal metabolism	MGI	Mouse	ENSMUSG00000021109
			Hif1a
abnormal intersomitic vessel morphology	MGI	Mouse	ENSMUSG00000021109
			Hif1a
abnormal interleukin secretion	MGI	Mouse	ENSMUSG00000021109
			Hif1a
abnormal heart ventricle morphology	MGI	Mouse	ENSMUSG00000021109
			Hif1a
abnormal heart tube morphology	MGI	Mouse	ENSMUSG00000021109
			Hif1a
abnormal heart looping	MGI	Mouse	ENSMUSG00000021109
			Hif1a
abnormal heart development	MGI	Mouse	ENSMUSG00000021109
			Hif1a
abnormal head mesenchyme morphology	MGI	Mouse	ENSMUSG00000021109
			Hif1a
abnormal embryonic epiblast morphology	MGI	Mouse	ENSMUSG00000021109
			Hif1a
abnormal embryo development	MGI	Mouse	ENSMUSG00000021109
			Hif1a
abnormal dorsal aorta morphology	MGI	Mouse	ENSMUSG00000021109
			Hif1a
abnormal developmental patterning	MGI	Mouse	ENSMUSG00000021109
			Hif1a
abnormal cytokine level	MGI	Mouse	ENSMUSG00000021109
			Hif1a
abnormal cardiac outflow tract development	MGI	Mouse	ENSMUSG00000021109
			Hif1a
abnormal blood vessel morphology	MGI	Mouse	ENSMUSG00000021109
			Hif1a
abnormal angiogenesis	MGI	Mouse	ENSMUSG00000021109
